# Bovine Polledness – An Autosomal Dominant Trait with Allelic Heterogeneity

**DOI:** 10.1371/journal.pone.0039477

**Published:** 2012-06-21

**Authors:** Ivica Medugorac, Doris Seichter, Alexander Graf, Ingolf Russ, Helmut Blum, Karl Heinrich Göpel, Sophie Rothammer, Martin Förster, Stefan Krebs

**Affiliations:** 1 Ludwig-Maximilians-University Munich, Munich, Germany; 2 Tierzuchtforschung e.V. München, Grub, Germany; 3 Laboratory for Functional Genome Analysis (LAFUGA), Gene Center, Ludwig-Maximilians-University Munich, Munich, Germany; 4 Göpel Genetik GmbH, Herleshausen, Germany; Huazhong Agricultural University, China

## Abstract

The persistent horns are an important trait of speciation for the family *Bovidae* with complex morphogenesis taking place briefly after birth. The polledness is highly favourable in modern cattle breeding systems but serious animal welfare issues urge for a solution in the production of hornless cattle other than dehorning. Although the dominant inhibition of horn morphogenesis was discovered more than 70 years ago, and the causative mutation was mapped almost 20 years ago, its molecular nature remained unknown. Here, we report allelic heterogeneity of the *POLLED* locus. First, we mapped the *POLLED* locus to a ∼381-kb interval in a multi-breed case-control design. Targeted re-sequencing of an enlarged candidate interval (547 kb) in 16 sires with known *POLLED* genotype did not detect a common allele associated with polled status. In eight sires of Alpine and Scottish origin (four polled versus four horned), we identified a single candidate mutation, a complex 202 bp insertion-deletion event that showed perfect association to the polled phenotype in various European cattle breeds, except Holstein-Friesian. The analysis of the same candidate interval in eight Holsteins identified five candidate variants which segregate as a 260 kb haplotype also perfectly associated with the *POLLED* gene without recombination or interference with the 202 bp insertion-deletion. We further identified bulls which are progeny tested as homozygous polled but bearing both, 202 bp insertion-deletion and Friesian haplotype. The distribution of genotypes of the two putative *POLLED* alleles in large semi-random sample (1,261 animals) supports the hypothesis of two independent mutations.

## Introduction

Since the beginning of animal husbandry, humans have tended to accumulate particular variations in domesticated animals. Thereby, the variants of practical interest for agriculture were selected as well as phenotypes that visibly distinguished particular animals. This practice was more frequent in pets (e.g. cats and dogs) but led to the fixation of some obvious breed characteristics in cattle, too [1]. The absence of horns (polled phenotype) as well as horn shape diversity represents such evident traits in cattle.

The persistent horn of the *Bovidae* consists of a pneumatised osseous core, which is fused with the frontal bone and covered by a cornified epithelium that grows outward from the skin at the base of the horn, thereby forming the cavernous visible horn. The development of horns is dependent on a number of different tissues and their interaction [2], [3]. Before the domestication of cattle, horns were important for the survival of the wild species. Even after domestication, horns were a desired trait (e.g. fixation and use as draught animals) in most cattle breeding areas until recently. Exceptions to that rule are found in some regions (e.g. Scotland and the Nordic countries) where polledness was a desired trait much earlier probably due to dense housing of animals during the long winter. Nowadays, commercial dairy or beef herds are mainly confined to barns or fenced-in enclosures such as pastures or corrals. Under these conditions horns are not only of little value but can lead to considerable economic loss due to a higher risk of injuries and the possible consequences (infection, carcass deterioration etc.). Therefore in modern cattle husbandry removing horns at an early age has become an accepted management practice. However, all used methods are debatable not least because of animal welfare implications [4]. Hence, breeding polled cattle may constitute a non-invasive option to replace the common practice by means of genetic selection.

In cattle, as in other *Bovidae*, horn development and morphology are characterised by a substantial degree of polymorphism. Even if only European cattle breeds (*Bos taurus*) are considered, there are completely hornless cattle breeds like Angus, and breeds with very short horns like Buša as well as breeds with very long and lyra-shaped horns like the Pannonian Podolian cattle [5]. The polled phenotype has been considered to be caused by a dominant inhibitor [3] that is characterised by the complete absence of corneous appendices [6]. In addition to diverse forms of cattle horns, abnormal types of horns called scurs frequently occur, i.e. incompletely developed horns that are not fused to the frontal bone [7]. Scurs can be considered as the intermediate phenotype between polled and horned because the horn core is not an outgrowth of the skull but originates from a separate ossification centre in the tissues located above the periosteum with subsequent fusion to the skull [2], [3].

While the inheritance pattern and mapping position of the *POLLED* locus on *Bos Taurus* Autosome 1 (BTA1) [8], [9], [10] is beyond controversy, the scurs locus displays heterogeneity [2] ^’^ [11] ^’^ [12]. To avoid complications due to possible interference between *POLLED* locus and type 1 [11] or type 2 [2] scurs syndrome we exclusively focussed on polled and horned animals of 31 cattle breeds of European origin. This material exploited the full capacity of the population structure of European cattle and in combination with high-density SNP genotyping and high-throughput sequencing, was used to identify the genetic variation that causes the polled phenotype in *Bos taurus*.

## Results

### The *POLLED* Locus Maps to Chromosome 1 in Divergent European Cattle Breeds

**Figure 1:**
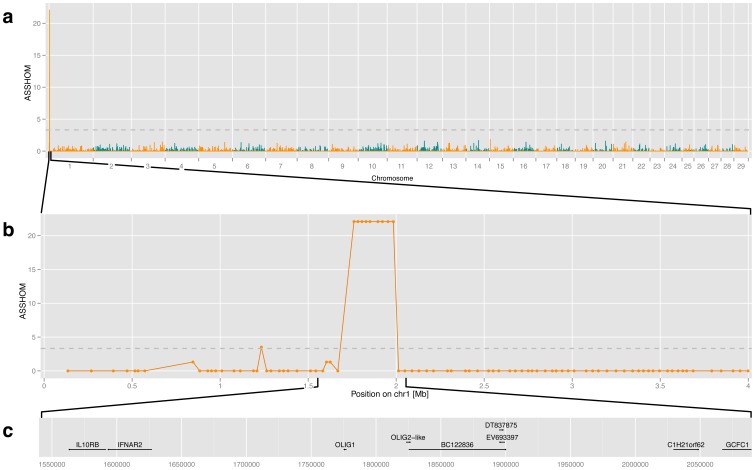
Homozygosity mapping in case-control design of polled and horned cattle animals. (**a**) Genome-wide association mapping of the *POLLED* mutation to the proximal end of bovine chromosome 1 (BTA1) with 162 affected animals and 162 controls (*asshom* method, Charlier *et al*. 2008). Nominal *asshom* statistic is presented chromosome by chromosome for all 29 bovine autosomes. Evidence for association (y axis) is measured as nominal *asshom* statistic with genome-wide significance (*P* = 0.0002, grey line) at 3.315, being determined by 50,000 nominal *asshom* statistics along chromosomes with randomly permutated markers. (**b**) Association mapping details for the region from 0 to 4 Mb on BTA1. Nine markers homozygous in all 162 cases covered a region from 1,760,113 to 1,983,902. Two informative SNP markers flanking this core (i.e. variable sites in the case group) are *ARS-BFGL-NGS-39992* (1,668,494 bp) and *ARS-BFGL-NGS-29653* (2,049,400 bp). (**c**) Candidate region of 547 kb (1.543–2.090 Mb (UMD3.1 genome build)) chosen for high-throughput sequencing and most likely encompassing the *POLLED* mutation. This interval is larger than homozygosity bracket detected by multi-breed design and nested five genes (IL10RB, IFNAR2, OLIG1, C1H21orf62 and GCFC1), one pseudo gene (OLIG2-like) and some not further annotated ESTs (e.g. BC122836, EV693397 and DT837875).

We collected DNA samples from twelve polled cattle breeds (Table S1). The applied mapping approach and major part (62%) of the samples has been presented in our previous study [10] and was now completed by additional 61 polled animals as well as by three additional polled breeds: Norwegian Red, Fjall cattle and Braunvieh. Case (homozygous polled, PP) and control (homozygous horned, pp) group each consisted of equal numbers of animals (162) from 18 breeds in total. As some breeds are consolidated either for the polled or horned phenotype uneven phenotype distribution was inevitable within some breeds. An improved case-control design accounting for this uneven distribution did not refine the mapping (Fig. 1). We mapped the *POLLED* locus in the same 381 kb interval (1.668 Mb - 2.049 Mb; UMD3.1 genome build) we already found in our previous study [10]. The genome-wide significance (*P*≤0.0002,) was determined by 50,000 random permutations of markers along chromosomes. The applied mapping procedure [13] performs searches for shared segments of homozygosity without the need of an identity by state and thus revealed two haplotypes. The most common haplotype (AGACAAGGA) was found in all but one case animals. Two copies of an alternative haplotype, with a variant allele at the sixth SNP (AGACA**G**GGA) were found in a homozygous polled Braunvieh bull (Fig. S1) that was one of the animals from the current addition to the mapping design. Genotyping of three related bulls revealed the same alternative haplotype associated with the polled phenotype in Braunvieh (Fig. S1). As the origin of polledness in Braunvieh is unknown and recombination within the homozygosity block of the available Braunvieh pedigrees could not be detected, the Braunvieh data did not refine the mapping.

In order to confirm and possibly improve the mapping results we embedded 89 heterozygous polled animals (*POLLED* carriers; Pp) and 62 horned relatives into the analysis. These 151 animals were genotyped with the same Illumina BovineSNP50 assay [14] and have not been used in the case-control design. These 89 Pp bulls comprise ten breeds and are ancestors or descendents of PP bulls included in the case group, except for carriers from the Witrug breed (WTG) (Table S1). The haplotypes were manually traced through the pedigree charts. The result clearly confirmed the mapping result (Fig. S1, S2, S3 and S4), but the absence of any observable recombination within the candidate region hindered further improvement of fine-mapping by linkage analysis.

### Sequencing Eight Polled and Eight Horned Sires did not Detected a Common Allele Associated with Polled Status

We selected seven PP, one Pp and eight pp bulls and performed high-throughput sequencing of a 547 kb interval (1.543–2.090 Mb (UMD3.1 genome build)) that nested the most likely location for the *POLLED* mutation [10]. The polled group was represented by four Holstein-Friesian (HF), two Galloway (GLW), one German Angus (DAN) and one Fleckvieh (FV) bull. One HF bull was declared as Pp while remaining seven bulls of polled group were progeny tested as PP. The horned group was represented by four HF, two FV, one Gelbvieh (FGV) and one Murnau-Werdenfelser (MWF) bull.

Paired-end sequencing of the libraries enriched for a 547 kb region on BTA1 resulted in average sequence depth of non-repetitive sequences of ∼40× per individual, with 98.9% of the non repeat-masked target region being covered. From these data, we identified 451 putative DNA sequence variants (DSVs), or an average nucleotide diversity of ∼0.15%. Further analysis of the sequencing data (on a locally installed instance of *galaxy* [15]) revealed almost complete absence of sequence variability in a region of 125 kb (1.697 to 1.822 Mb) between all 16 sequenced animals. Under the assumption that the *POLLED* locus is biallelic with dominant inheritance [7], [9] and because the reference sequence (RefSeq) is based on a horned Hereford cow [16], the causative variant has to be homozygous in the seven PP sires, heterozygous in one Pp sire and not present in RefSeq or in the eight pp sires. Applying this filter to the 451 DSVs did not detect a common allele associated with polled status. Also a visual inspection in a genome browser did not yield DSV concordant with the *POLLED* genotypes. These results suggested at least two plausible explanations: (i) the targeted 547 kb interval that was covered to 98.9% for the non repeat region harbors the functional mutation(s) in the remaining 1.1% (ii) some of DSVs are causal but there is allelic heterogeneity at the *POLLED* locus and different alleles have been selected in different geographic regions or breeds.

### Dividing Sequenced Animals into Two Groups Suggests Allelic Heterogeneity of the *POLLED* Phenotype

**Figure 2:**
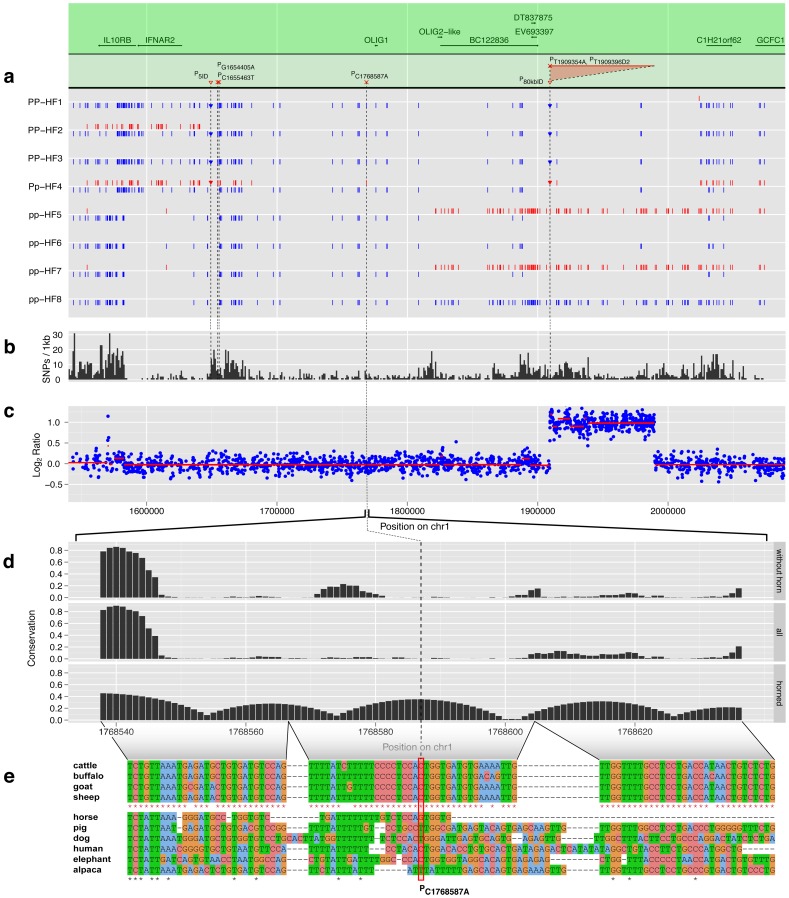
DNA sequence variants in Polled and horned Holstein Friesian. (**a**) Positions of DNA sequence variants (DSV) detected in the eight HF animals are presented by bars for SNPs and inverted triangles for InDels, with red symbols for heterozygous and blue symbols for homozygous differences from the reference sequence. Homozygous polled bulls are PP-HF1, PP-HF2 and PP-HF3. Pp-HF4 is declared as heterozygous polled and pp-HF5, pp-HF6, pp-HF7 and pp-HF8 are horned. Annotated genes and ESTs lying in the re-sequenced region are shown in the green shaded region. The five candidate mutations for polledness are superimposed: three SNPs were outlined by red crosses, 5 bp InDel and the duplicated region P_80kbID_ were highlighted by inverted triangle. The red triangle area above P_80kbID_ marks the duplicated sequence. (**b**) Within-species conservation. The nucleotide diversity in the re-sequenced region is shown by a density plot of bovine variants from dbSNP per kb. (**c**) Copy number variations. The ratio of mapped sequence reads between polled and horned animals is plotted with blue dots. The red line represents the result of segmentation analysis, showing the average ratio in the determined bins. (**d**) Across-species conservation. For each candidate mutation the surrounding base conservation for species without horn, bovid species and all aligned species was determined as *PhastCons* score calculated from *multiz* alignments. As an example, here we display the base conservation for the candidate mutation P_C1768587A_. (**e**) The underlying multi-species alignment for the across-species conservation calculation for the candidate region is shown in plot d. The candidate mutation P_C1768587A_ is highlighted within a red frame and stars in red and black indicate identity among bovid and all animals, respectively.

Eight of 16 sequenced sires are Holsteins and in the case of the allelic heterogeneity these could most probably harbour the same *POLLED* mutation. The average sequence depth of non-repetitive sequences in eight HF bulls was ∼29× per individual, covering 98.5% of the non repeat-masked target region. From these data, we identified 312 putative DSVs, or an average nucleotide diversity of ∼0.11% (Fig. 2). Applying the same consecutive filtering as above reduced the number of potential causal variants to seven. On the proximal end of the region homozygous in polled HF (1.648–2.027 Mb; Fig. 2) we detected a complex InDel event, replacing 7 bp (cgcatca; RefSeq: 1,649,163–1,649,169) by 12 bp (ttctcagaatag) and thus resulting in a 5 bp longer sequence in polled HF animals (allele P_5ID_). On the distal end of the region of homozygosity in polled HF we detected a duplication of an 80,128 bp (1,909,352–1,989,480 bp) sequence (Fig. 2). This large sequence is seamlessly duplicated in the same direction and only differs from the RefSeq by one T→A transversion at the third position after the beginning of the duplicated sequence (P_T1909354A_) and by a two-base pair (TG) deletion at the 45^th^ position in the duplicated sequence. This 2 bp deletion corresponds to position 1,909,396 in the original sequence and will be denoted as P_1909396D2_. Both variants close to the junction between the original and the duplicated sequence, P_T1909354A_ and P_1909396D2_, allow for a PCR design for the detection of this 80 kb InDel (allele P_80kbID_). In addition to P_5ID_ and P_80kbID_, both flanking the homozygosity region in polled HF, we detected five point mutations at the positions 1,654,405 (G→A), 1,655,463 (C→T), 1,671,849 (T→G), 1,680,646 (T→C) and 1,768,587 (C→A), respectively. In these five candidate SNPs (P_G1654405A_, P_C1655463T_, P_T1671849G_, P_T1680646C_, P_C1768587A_) mutant alleles determine the sequence in the polled HF animals. These seven candidate mutations do not include any known coding sequence, or splice site, or intronic region, or any known regulatory elements (Fig. 2). Furthermore, none of these variants were previously reported in dbSNP (http://www.ncbi.nlm.nih.gov/projects/SNP/).

**Figure 3:**
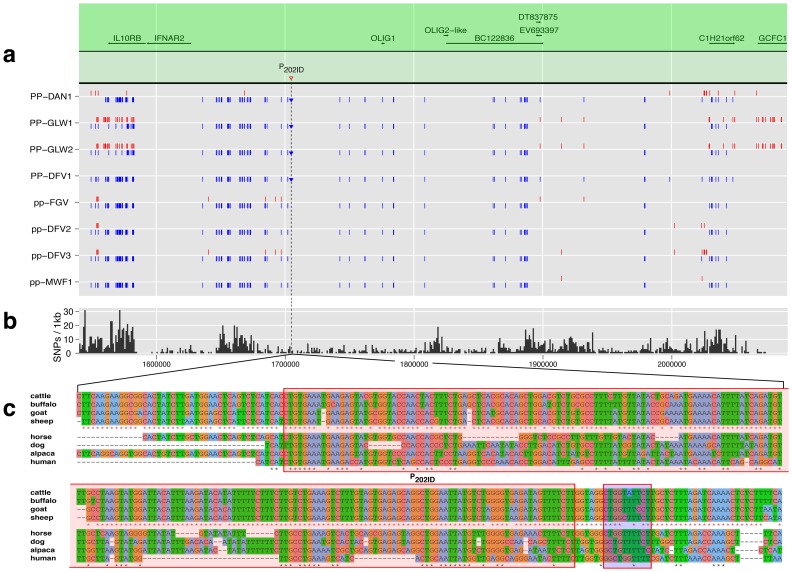
DNA sequence variants in diverse polled and horned cattle breeds. (**a**) SNP genotypes. The genomic position of DNA sequence variants (DSV) called by high-throughput sequencing of the target region are displayed as bars (SNPs) or triangles (InDels), in blue (homozygote) and red (heterozygote). Homozygous polled bulls are PP-DAN1, PP-GLW1, PP-GLW2 and PP-DFV1 and horned bulls are pp-FGV, pp-DFV2, pp-DFV3 and pp-MWF1. The candidate duplication P_202ID_ is highlighted by an inverted triangle. The annotated genes and ESTs in this region are displayed in the green area. (**b**) Within-species conservation. The bovine variant density from dbSNP is shown in the re-sequenced region per kb. (**c**) Multi-species alignment. The genomic multi-species alignment around the candidate duplication P_202ID_ was built with *multiz*. Sequence identities among bovid animals are displayed with red stars whereas black stars denote the identity among all animals. The duplicated region is highlighted by the red shaded area and replaces the blue shaded nucleotides in polled animals.

The remaining eight non HF sires (four PP and four pp) belong to spatial and genetic quite differentiated breeds: Angus, Galloway, Fleckvieh, Gelbvieh and Murnau-Werdenfelser. Nevertheless, both PP and pp sires were homozygous for the common haplotype that is always homozygous in PP animals [10]. Considering these eight sires separately paired-end sequencing identified 248 putative DSVs, or an average nucleotide diversity of ∼0.08%. Applying the same filter to the 248 DSVs yielded only one complex insertion-deletion (InDel) event as the putative causative variant (Fig. 3), which was confirmed by visual inspection in a genome browser to be the only DSV concordant with the pp and PP genotypes. The sequence of 212 bp (1,705,834–1,706,045 bp) is duplicated and replaces a sequence of 10 bp (1,706,051–1,706,060 bp). This InDel (P_202ID_) is between the genes *IFNAR2* and *OLIG1* (Fig. 3). The InDel P_202ID_ again does not disrupt any known coding sequence or a splice site, or an intronic region, or any known regulatory regions. All candidate DSVs detected in HF subset were homozygous for RefSeq allele in all eight non HF sires, both in PP as well as in pp.

### Target Genotyping of Candidate DSVs in Polled Animals, Random Samples and Diversity Panel Supports Allelic Heterogeneity of the *POLLED* Phenotype

In order to further investigate the presumed allelic heterogeneity of *POLLED*, we chose sperm samples of polled bulls of all of the most important cattle breeds with European origin. This comprised 89 Pp bulls of ten breeds (Table S1). Moreover, we complemented the sample of the homozygous case group by additional PP bulls of Holstein-Friesian (HF, including Red-Holsteins (RH)), German Fleckvieh (FV) and Jersey (JY).

The P_202ID_ allele was in complete LD to the *POLLED* allele in all breeds but HF, JY and WTG which turned out to carry the P_202ID_ allele only sporadically. Polled HF, JY and WTG animals declared as Pp but missing the P_202ID_ allele were carriers of the haplotype block of seven candidates detected by sequencing of HF animals (P_5ID_, P_G1654405A_, P_C1655463T_, P_T1671849G_, P_T1680646C_, P_C1768587A_ and P_80kbID_). Furthermore, HF and JY animals declared as PP and missing P_202ID_ were carriers of two copies of the haplotype detected by sequencing of polled HF animals.

**Figure 4:**
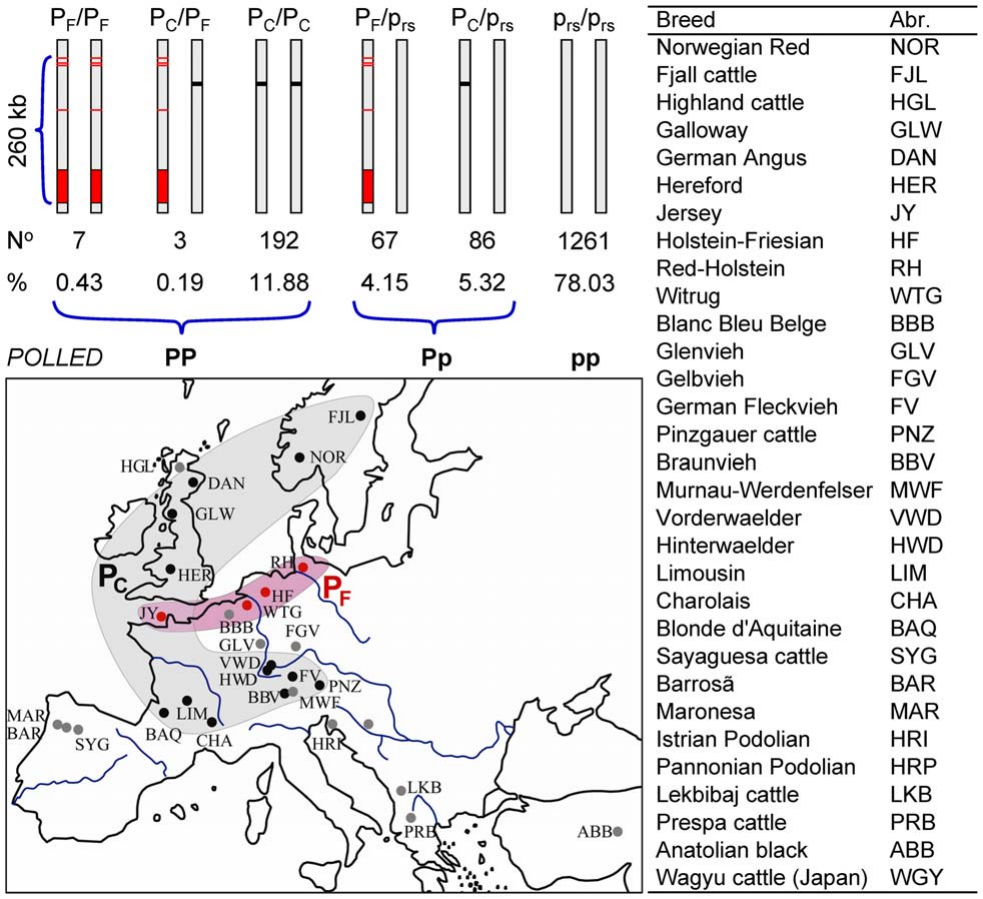
Distribution of *POLLED* candidate variants in European cattle breeds. The largest proportion (shown in the row marked %) of genotyped animals (78.03%) were homozygous for the wild-type allele at all six candidate variants of the *POLLED* locus, i.e. they inherited two copies of the RefSeq haplotype p_rs_ presented by vertical gray bar. Plausibility analyses suggested all these 1261 (shown in the row marked with N^o^) p_rs_/p_rs_ animals as being horned, therefore pp at the *POLLED* locus. Five candidate mutations detected by sequencing of polled and horned Holstein-Friesian animals form a haplotype block (P_5ID_­P_G1654405A_­P_C1655463T_­P_C1768587A_­P_80kbID_) consisting of three SNPs flanked by two InDels and called P_F_ signifying polledness of Friesian origin. The five candidate mutations of Friesian polledness were superimposed on RefSeq background (vertical gray bar): three SNPs and a 5 bp InDel were outlined by red horizontal bars and the duplicated region P_80kbID_ by the red area. The P_F_ block is in perfect association with *POLLED* genotype and segregate only in cattle breeds originating from north-western coast of continental Europe (HF, RH, JY and WTG). The candidate mutation P_202ID_ is represented by black horizontal bar on RefSeq background. All animals carrying one (86) or two copies (192) of the P_202ID_ are polled (Pp or PP, respectively) and belong to breeds originating from Scandinavia, Great Britain, France and South-Germany, hereafter figuratively called polledness of Celtic origin, P_C_. Three bulls with PP genotype determined by extensive progeny testing were found to be heterogeneous polled at candidate gene level, i.e. P_C_/P_F_. The genotyping of their sampled relatives as well as entire experimental design provide no evidence for recombination within P_F_ haplotype block. The geographic origin of the sampled breeds is outlined by dots on Europe map. Breeds lacking polled samples are represented by gray dots. The breeds with P_202ID_ as only causal variant or variant in perfect association with *POLLED* locus are marked by black dots. The breeds with P_F_ as predominant variant in perfect association with *POLLED* locus are marked by red dots. The approximate distribution area of Celtic and Friesian polledness are highlighted as gray and red shading respectively.

To further proof the hypothesis of allelic heterogeneity of *POLLED*, we randomly sampled 400 animals in South-Germany complemented by random samples from target breeds. This semi-random sample comprised sets of 238 random HF/RH, 293 HF-FV crosses, 52 random JY, 50 random PNZ, and 211 animals from our cattle diversity panel (Table S1). These 1,262 DNA samples as well as the case and the carrier group were chosen for genotyping for the candidate variants: P_202ID_, P_5ID_, P_80kbID_ P_G1654405A_, P_C1655463T_, P_T1671849G_, P_T1680646C_, and P_C1768587A_. Two SNPs P_T1671849G_ and P_T1680646C_ were excluded due to sporadic and solitary occurrence of the candidate mutation on RefSeq background in horned animals. So far, we do not observe recombination within P_F_ haplotype block (from P_5ID_ to P_80kbID_) and consequently do not exclude any candidate mutations by recombination. As soon as one candidate was excluded by confirmed genotyping we cancelled further genotyping of the respective marker. The breeding records, including polled certificates of the genotyped animals and their ancestors as well as their breed affiliation, were used to confirm the congruence between the obtained genotyping results at six remaining candidate mutations and polled status. The results are presented in Fig. 4 and summarised below. The single candidate variant P_202ID_ detected by the sequencing of eight non HF sires and the block of five candidates detected by sequencing of eight HF animals (P_5ID_, P_G1654405A_, P_C1655463T_, P_C1768587A_ and P_80kbID_) segregate independently without evidence for any interference or recombination. As all animals carrying one or two copies of the P_202ID_ allele are polled (Pp or PP, respectively) and belong to breeds originating from geographical areas with Celtic culture [17] we hereafter figuratively and provisory called this allele P_C_, indicating polledness of Celtic origin (see Fig. 4). Appropriately, the haplotype block (260 kb long) detected by sequencing of HF animals is hereafter called P_F_, indicating polledness of Friesian origin. Both P_C_ and P_F_, independently and in combination, were in perfect association with *POLLED* locus. There were two HF and one JY bull declared as PP which we found to be heterogeneous polled at candidate level, i.e. P_C_/P_F_. These three bulls were progeny-tested and mated to a large number of horned cows, permitting an official declaration as PP by the respective breeder associations. The pedigrees chart and genotype analyses of the P_C_/P_F_ bulls and their sampled relatives (Fig. S3 and S4) clearly support allelic heterogeneity at the *POLLED* locus. Our diversity panel completed the semi-random sample (Table S1) and both together cover the European cattle population from the domestication centre, over the most likely dispersal routes for this species to the western- and northernmost parts of Europe (Fig. 4). This spatial well distributed panel comprises breeds with very small horns as well as extremely large horns [5] and with high genetic diversity between as well as within breeds [18]. In these horned breeds, only wild-type alleles (p_rs_) were detected for all six candidate mutations, hindering further sieving of causative variants from passenger variants in this design.

### Sequence Conservation Among Horned Ruminants and Other Mammals Allows Scoring of Candidate Causal Mutations

The DSVs P_G1654405A_ and P_5ID_ are part of repeat-masked regions (MLT1B and LTR13BT), hence no conservation score (*PhastCons* scores) could be computed. Local blat searches against the selected species revealed only short multiple alignments to various genomic locations; in consequence no local alignments could be built. This however makes it unlikely that these two DSVs are linked to horn development as the flanking sequence is not conserved among horned ruminants. Moreover, these DSVs are found in a region with a high density of SNPs (Fig. 2B), indicating little selective pressure and making it less likely to encode for a functional element important for horn development.

The sequence flanking P_C1655463T_ has a low conservation score, even among ruminants, and the local alignment (Fig. S5) shows that the corresponding sequence in the goat genome harbours a T, the same base as the variant allele found in polled Holstein cows. Thus, P_C1655463T_ is less likely being causative for the polled phenotype.

The RefSeq nucleotide C at SNP P_C1768587A_ is conserved only among bovid ruminants, where the variant allele is at the central position of a 24 nucleotide block that is identical in all four available *Bovidae* genomes. Also, the surrounding sequence is highly conserved among *Bovidae* but far less among other mammals (Fig. 2D and E). The SNP density of the whole region (Fig. 3B and 3C) including P_C_ shows a contiguous area with high sequence conservation within cattle, making it a likely candidate for the presence of an un-annotated functional element.

Both P_T1909354A_, and P_1909396D2_, nested within the 80 kb duplication, affect elements that are conserved only among the bovid ruminants (Fig. S6). However, the 80 kb duplication can be causative itself, and sequence conservation of single elements within these 80 kb does not necessarily reflect the consequence this duplication may induce.

Sequence conservation across and within species thus allows ranking of the candidate mutations within P_F_ haplotype, resulting in the P_80kbID_ duplication (including the two variations P_T1909354A_ and P_1909396D2_) and the SNP P_C1768587A_ as two most plausible candidates. The candidate P_C1655463T_ is considered as least likely as it coincides with sequence variation within different members of the *Bovidae* family suggesting a lack of functional constraint at this position.

## Discussion

The polled phenotype in cattle has been a target of selection and research since long. Large economic value of the phenotype [19], the simple dominant inheritance known since 1936 [6] and the mapping position known since 1993 [9] provided hope for an early and easy detection of the causal variant. This, however, was not the case due to special features of the phenotype, underlying causal mutations and livestock genetics: (i) the identification of functional candidate genes by the usual comparative trajectory with humans or mouse is hardly possible for this phenotype specific to *Bovidae*, (ii) the lack of functional and even positional candidate genes in large fractions of candidate region hindered identification of mutations conferring the polled phenotype and (iii) possible allelic heterogeneity of the phenotype investigated in a highly mobile species (world-wide and across-breeds use of some founders by artificial insemination) can reduce the ability to exploit haplotype diversity between breeds.

In this study, we used approaches beyond LD-based fine mapping to reduce the list of candidate causative genes without relying on any *a priori* assumptions of gene function. According to the contemporary trajectory of fine-mapping by a relatively dense SNP marker panel, high-throughput re-sequencing and association proven in a large panel of individuals sampled across the European continent we detected a single complex insertion-deletion event (P_202ID_) perfectly associated with the *POLLED* gene in most European cattle breeds. The absence of any other congruent candidate variant in the entire chromosome segment detected by fine-mapping, as well as perfect association with the phenotype, high sequence conservation of the candidate fragment among horned ruminants and absence of the appropriate conservation in un-horned mammals (Fig. 3C), clearly suggest P_202ID_ as most probably being the causal mutation for polledness in most *Bos taurus* breeds. The fact that InDel P_202ID_ resides in a region without known function is neither a clear argument against nor in favour of the candidate but is rather a proof of current rudimentary knowledge about gene function or presumed effects of candidate causal variants. The plausible causal mutation must fall within the limits of the dominant mode of inheritance. One possibility is haploinsufficiency which unlike in type 2 scurs [2] should not result in an enhancement of the effect in homozygotes. The next possibility is a gain-of-function mutation which would be characterized by a complete inhibition of the separate ossification centre in the tissues above the periosteum [3]. Such new regulatory elements with abnormal function may fall within the class of non-coding RNA and micro RNA (miRNA) or promoter sequences. However, their *de novo* prediction from un-annotated sequence of a non-model organism is highly error-prone and was not used for speculation about the mechanisms causing polledness.

The biology of horn development has not been studied in detail, partly also due to the obvious lack of model organisms. The development of a new ossification centre should probably engage the well characterized network of bone development with the *RUNX2* master transcription factor and its known regulators including twist proteins [20] and several miRNAs [21]. Indeed, mapping of the type 2 scurs locus has identified a *TWIST1* mutation as likely cause [2] and expression analysis of skin biopsies of the horn bud area in newborn polled and normal calves has shown evidence of endothelial-mesenchymal transition [22], a process involving bone morphogenetic protein signalling and twist protein activity. Genetic polledness is also known in goat and sheep, but the underlying mutations are mapped to chromosomes without synteny to the bovine polled locus investigated, here. Moreover, the polled/horned phenotype shows sexual dimorphism in sheep [23] and in goat is even linked with impaired sexual development [24], so that it is assumed that polledness in these species is caused by different mechanisms.

Including the cattle breeds originating from the North See area –HF, JY and WTG– led (Fig. 4) to the detection of a single haplotype block composed of three SNPs flanked by two InDels, all possible candidate causal variants for an independent mutation causing polledness. This Friesian haplotype, P_F_, segregates independently from the previously mentioned Celtic mutation, P_C_. Both are complementary and there is no evidence for any interference or recombination. Two Friesian and one Jersey bull were heterogenic at the *POLLED* locus (P_F_/P_C_) and declared as homozygous polled by progeny testing, i.e. all progeny from mating with horned dams are polled. All three bulls were intensively used for artificial insemination without any particular features and bred hundreds of exclusively polled offspring. Recombinant offspring of these bulls can supply important information for a formal test of allelic heterogeneity of the *POLLED* phenotype.

Not only economical reasons but also increasing serious animal welfare issues urge for a solution in the production of hornless cattle other than by dehorning. Even though the positive effects of genetic dehorning predominate, possible negative aspects should not be dismissed. In spite of a strong selection and serious attempts to improve breeding value of the polled sires these still lag behind in performance. For example, the best progeny tested polled (Pp) Fleckvieh bull currently ranks at position 1,724 of selection index, more than 1.3 standard deviations worse than the top 100 bulls. We find a similar situation in the Holstein breed. The inferior dairy breeding values of polled bulls might be caused simply by long-standing neglect of polled animals within sophisticated breeding programs or by multiple pleiotropic effects. It is known that some domestication traits result from mutations that cause large phenotypic effects but include deleterious pleiotropic effects [25]. Short-termed agricultural interests can obviously overcome the negative pleiotropic effects of some large phenotypic effects like in *myostatin* null mutations in cattle [26]. The recently mapped type 2 scurs syndrome locus with its underlying most probably causal mutation in the *TWIST1* gene [2] represents another breeder-selected trait that is negatively correlated with fitness due to embryonic lethality for the homozygous state. To avoid possible negative long-term impacts, possible deleterious pleiotropic effects should be closely investigated before the massive amplification of the *POLLED* gene in large cattle populations.

In conclusion, we describe the mapping as well as the perfect association of one InDel and one short haplotype causing polledness in cattle and suggest the conservation score as prioritising criterion of the most probable causal mutations. The distribution of the two putative *POLLED* alleles across the European continent supports the hypothesis of two independent mutations. Traits with allelic heterogeneity are no exception but tedious to decipher. We presented research strategies which could be more widely applicable for deciphering the molecular mechanisms of phenotypes with allelic heterogeneity.

## Materials and Methods

### Ethics Statement

Collection of blood samples was conducted exclusively by local veterinarians during regular health inspection and quality control of breeding records on the farms, so that randomness of the sample was assured and no ethical approval was required for this study. The regular health inspection includes annual IBR diagnostics (BGBl. I Nr. 74 S. 3520 ff) and ruminant metabolisms control at Lehr- und Versuchsgut, Oberschleißheim, Germany (Dec. 55.2-1-54-2531.3-80-10). The regular quality control of breeding records includes paternity testing organised by the respective breeding associations. Paternity testing involves blood, semen and hair root samples. Blood sampling by veterinarians with state examination avoids unnecessary pain, suffering and damage and is in accordance with the German Animal Welfare Act. Semen samples were collected by approved commercial artificial insemination stations as part of their regular breeding and reproduction measures in cattle industry. Hair roots samples were collected by breeders themselves (hair plucking from pinna or tail-tassel) as part of regular sampling for parentage control. As acknowledged below and specified in Table S2 we used these regularly sampled blood, semen and hair roots samples for DNA preparation necessary for this study.

### Animals

All animals sampled and genotyped in this study are presented in Table S1 according to their breed affiliations and purpose in the experimental design. These 1,675 animals originate from 31 European cattle breeds belonging to the *Bos taurus* subspecies. The DNA samples of all 162 cases, 162 controls and 89 carriers were genotyped genome-wide with the Illumina BovineSNP50 BeadChip [14]. All 162 cases, 89 carriers and 1,262 semi random animals were genotyped for the revealed candidate mutations.

### Phenotypes and Declaration of the Underlying *POLLED* Genotype

Polledness or complete absence of horns is a visible phenotype that can be identified at relatively young animals (four to six months). Because the growth of scurs occurs later in life than horns, phenotyping of some scurred animals will not be possible until nine to eighteen months of age [12]. Polled animals, especially breeding bulls and important dams, are declared as polled (name suffix P) in their pedigree certificate and/or other records. One P designates polled animals with the PP or Pp genotype at underlying *POLLED* locus. Breeding animals with 12 to 15 (depending on breeding organisation) consecutively polled offspring originating from horned mates are declared as homozygous polled and get a name suffix PP. One horned offspring with confirmed paternity is sufficient to declare a polled animal as Pp.

### Homozygosity Mapping

DNA samples of 162 case and 162 controls were genotyped with the Illumina BovineSNP50 BeadChip [14]. Marker order was based on release UMD3.1 of the *Bos taurus* genome (http://www.cbcb.umd.edu/research/bos_taurus_assembly.shtml). The SNP haplotypes were inferred and missing genotypes imputed using hidden Markov models (software package *Beagle* [27]). Three cohorts, namely trios (two parents, one offspring), pairs (one parent, one offspring) and unrelated animals were formed, including those animals that turned out not to be relevant for this study (2,721 animals in total). Genome-wide homozygosity mapping in polled animals and controls was performed using the *asshom* procedure [13]. Case and control genotypes served as input data after completion of haplotype-inference and imputation by the *Beagle* package. To determine the statistical significance of each summary score, we permutated (50,000 permutations) the complete markers along the chromosomes [10] and estimated the summary score as harmonic mean across all cases. Thus, the corresponding *P* values were corrected for multiple testing and accounted for the level of inbreeding within the cases [13]. The statistical significance based on 50,000 permutations is comparable to the number of markers used.

### Genotyping of the Candidate Causal Mutations

In general, 30–60 ng of genomic DNA were used for genotyping of the candidate mutations. InDel variants P_202ID_ and P_5ID_ were PCR amplified (94°C 30 sec, 58°C 60 sec, 72°C 60 sec for 31 or 35 cycles, respectively) using primer binding sites flanking the InDel events (5′-TCAAGAAGGCGGCACTATCT-3′ and 5′-TGATAAACTGACCCTCTGCCTATA-3′ for P_202ID_ and 5′-FAM-CCTTGTCACGTTAGATGTATGTCC-3′ and 5′-TCAATCTCTAATAAGGAACAGAAGAAA-3′ for P_5ID_). PCR products were size-separated and visualized by 2% ethidium-bromide stained agarose gel electrophoresis (P_202ID_) or analysed on an ABI Prism® 3130*XL* DNA sequencer (P_5ID_).

Genotyping of the P_80kbID_ was performed by use of two primers flanking the variable site P_1909396D2_ (5′-GAAGTCGGTGGTCTGAAAGG-3′ and 5′-TGTTCTGTGTGGGTTTGAGG-3′). PCR amplification (32 cycles 94°C 30 sec, 59°C 60 sec, 72°C 60 sec) resulted in a RefSeq related product (p_rs_) which was obtained in all animals, whether horned or polled. Additionally, a second P_F_ related product, containing the two-base pair (TG) deletion was observed in all animals bearing one or two copies of the P_F_ haplotype. Discrimination of the two products differing 2 bp in size was performed on an ABI Prism® 3130*XL* DNA sequencer. Animals bearing P_F_/P_F_ were distinguished from P_F_/p_rs_ by quantitative evaluation of the obtained signals. P_F_/P_F_ animals yielded signals of similar peak heights for the P_F_ and p_rs_ products, while a heterozygous constellation (P_F_/p_rs)_ resulted in signal intensities of approximately double height for the p_rs_ product when compared to the P_F_ specific product.

SNP variants P_G1654405A_ and P_C1655463T_ were genotyped by competitive allele-specific PCR using commercially available kits (KASPar®, KBioscience). PCR was performed as recommended by the manufacturer, while subsequent discrimination of the obtained fluorescent-labelled (FAM and CAL Fluor Orange 560) allele-specific products was performed on an ABI Prism® 3130*XL* DNA sequencer.

SNP variant P_C1768587A_ was analysed by PCR-RFLP. Genomic DNA was amplified (32 cycles 94°C 30 s, 58°C 60 s, 72°C 60 s; 5′-CTGGAACCACGGATTACACAG-3′ and 5′-ACAGTTATGGTCAGGAGGCAAA-3′). Subsequently, three µl of the PCR product were treated with three units of TspRI (65°C for a minimum of 3.5 h). Obtained fragments were size-separated and visualized by 2% ethidium-bromide stained agarose gel electrophoresis.

### Targeted re-sequencing

One µg of Genomic DNA was randomly sheared by sonication (Bioruptor, Diagenode, Liege, Belgium) for 25 cycles (30 sec on/off, “low” intensity). Sheared DNA with a median size of 200–300 bp was rendered blunt-ended and 5′-phosphorylated (NEBnext end repair module, New England Biolabs Inc, Ipswich, USA). After addition of a single non-templated 3′-A (NEBnext A-tailing module) fragments were ligated to Illumina compatible adapters that carried a sample-specific 3 nt barcode and a 3′-T overhang. The ligated library was size-selected on a 2% agarose gel and amplified by PCR with Illumina PE1 and PE2 primers. Equimolar amounts of the eight samples were pooled for array-capture (Agilent 244 k capture Array, Agilent, Santa Clara, USA; custom designed by e-array, repeat-masked, 3 bp tiling). Briefly, the libraries were hybridised for 65 h at 65°C, washed and eluted with nuclease-free water for 10 min at 95°C. The eluted DNA was concentrated in a vacuum centrifuge, amplified by PCR (10 cycles 98°C 15 s, 65°C 30 s, 72°C 30 s) and purified with Ampure XP beads.

### Mapping and Variant Calling

Sequence reads from the Illumina Genome Analyzer IIx were aligned to the bovine reference genome (UMD3.1) using *BWA* [28]. The mapped reads were filtered for PCR duplicates with *SAMtools* [29] and only uniquely mapping reads were retained. A pileup of the mapped reads was created for each animal using *SAMtools* and variants were detected with *VarScan* (v.2.2.7) [30] at a minimal coverage of 20 and a minimal variant frequency of 0.01.

The applied filters for the detection of potentially causative variants were: Homozygosity in the PP sires with a variant frequency range of 95 to 100%. For the heterozygote carrier Pp they must fall within a range of 40 to 60%, while the resulting candidate variants must be undetected in the pp sires. The putatively causative variants must be different from the UMD3.1 genome sequence, which was derived from a horned Hereford dam and therefore cannot carry the polled allele.

### Copy Number Variation

For the identification of large insertion-deletion events between polled and horned animals we calculated the coverage ratio between the two groups in dynamical bin sizes. Each bin size was calculated by iterating over the mapped reads in the reference group until a determined number of reads (400) was reached and the start position of the last read was taken as bin size [31]. This step was repeated until the whole target region was processed. Then, for the target group the number of reads falling in each bin were counted and used for the calculation of the log2 ratios between reference and target group. The log2 ratios were plotted using R [32] and the average ratios were segmented using the circular binary segmentation implementation in the *DNAcopy* package (v1.14.0) from *Bioconductor* [33].

### Multi-species Alignment and Conservation Scores

For the re-sequenced target region (chr1:1,543,412-2,089,648) we created multiple alignments for bovid ruminants (cattle, sheep and water buffalo), for unhorned mammals (horse, dog, human, mouse, pig, elephant, alpaca and dolphin) and for all selected species. The repeat-masked bovine genome sequence of the target region was obtained from UCSC genome browser [34] and used as reference for each of the pair-wise *lastz* genome alignments [35]. The resulting alignments were chained and netted to obtain best matching hits [36] and multi-species alignments were generated with *multiz* [37] projected to the bovine reference. Additionally, short read sequences from the goat genome project (SRA Accession number: SRX016522) were added to the multi-species alignments only in the neighborhood of candidate mutations. To do so we aligned the short read to the bovine reference using *blat* [*38*] and manually selected the best alignments to the bovine reference.

The reference genomes of sheep (*Ovis aries* 1.0), human (hg19), mouse (mm9), dog (canFam2), horse (EquCab2) and pig (Sscrofa9.1) were obtained from the FTP-site at UCSC. The draft genome sequences from alpaca, elephant and dolphin were obtained from the Broad Institute [39] and the water buffalo genome sequence was taken from the Indian Buffalo Genome Project (http://210.212.93.84).

From the multiple species alignments we calculated the base conservation among horned animals, unhorned mammals and for all species using the *phast* package. For that purpose, we estimated a phylogenetic tree with *PhyloFit* from which the conservation score for each base was calculated with *PhastCons*.

## Supporting Information

Figure S1 **The pedigree chart of most polled Braunvieh.** All declared polled Braunvieh bulls are descendents of the well-known American Brown-Swiss bull BS1 which is founder of polledness in Brown-Swiss/Braunvieh cattle population. The case individuals (PP) are represented by solid circles (females) and squares (males); declared carriers by half-filled symbols; not sampled individuals are marked with a diagonal line. The haplotype associated with polledness (red letters) of the four genome-wide genotyped bulls can be traced back to the same carrier bull BS1. This pedigree includes also four polled Braunvieh animals (*) genotyped only for candidate mutations. All sampled PP animals were genotyped as P_C_/P_C_ and all Pp as P_C_/p_rs_.
(TIF)

Figure S2 **The pedigree chart of all sampled Fleckvieh bulls composing case and carrier group in Table S1.** The case individuals (PP) are represented by solid circles (females) and squares (males); declared carriers by half-filled symbols; not sampled individuals are marked with a diagonal line. To reduce complexity of the pedigree not all relationships were presented. At two positions (A and B) there are relationships to important sires indicated. The inbreeding of the Fleckvieh bull (PP-DFV1) chosen for re-sequencing is superimposed by red lines. All sampled Fleckvieh PP animals bear two copies of the common haplotype (AGACAAGGA) and were genotyped as P_C_/P_C_. All Pp bear one copy of the common haplotype and were genotyped as P_C_/p_rs_. No recombination was detected in the common haplotype.
(TIF)

Figure S3 **The pedigree chart of all sampled Holstein bulls composing case and carrier group in Table S1.** The case individuals (PP) are represented by solid circles (females) and squares (males); declared carriers by half-filled symbols; not sampled individuals are marked with a diagonal line. To reduce complexity of the pedigree not all relationships were presented. At positions A, B and C there are relationships to important sires indicated. The founder of the Celtic polledness is indicated by D. The carriers and cases of Friesian and Celtic polledness are filled with gray (genotyped as P_F_/p_rs_ and P_F_/P_F_) and black color (genotyped as P_C_/p_rs_ and P_C_/P_C_), respectively. Two heterogeneous polled bulls were genotyped as P_C_/P_F_. At the SNP-Chip level all sampled Holstein PP and Pp animals bear two or one copy of the common haplotype (AGACAAGGA) and there was no recombination detected.
(TIF)

Figure S4 **The pedigree chart of all sampled case and carrier Jersey bulls**. The case individuals (PP) are represented by solid circles (females) and squares (males); declared carriers (Pp) by half-filled symbols; not sampled individuals are marked with a diagonal line. The founder of the Friesian polledness in Jersey breed is indicated by F. The carriers and cases of polledness with Friesian and Celtic origin are filled with gray (genotyped as P_F_/p_rs_ and P_F_/P_F_) and black color (genotyped as P_C_/p_rs_ and P_C_/P_C_), respectively. Two heterogeneous polled bulls were genotyped as P_C_/P_F_. At the SNP-Chip level all sampled Jersey PP and Pp animals bear two or one copy of the common haplotype (AGACAAGGA) and there was no recombination detected.
(TIF)

Figure S5 **Across-species conservation for variant P_C1655463T_.** (**a**) The sequence conservation around the position of the candidate mutation P_C1655463T_ among animals without horn, horn-bearing and among all animals are represented as *PhastCons* scores, calculated from the underlying multi-species alignment in plot b. (**b**) Sequence identity among horn-bearing animals is denoted with red stars and black stars are used for identity in all aligned species. The position of the candidate DNA sequence variant is outlined by a dashed black line and is highlighted in the multi-species alignment by a red frame.
(TIF)

Figure S6 **Across-species conservation for variants P_T1909354A_ and P_1909396D2_.** (**a**) The *PhastCons* conservation scores of the sequence around the two DNA sequence variants P_T1909354A_ and P_1909396D2_ are shown. Both variants are outlined with black dotted lines. (**b**) Sequence identity is marked in the multi-species alignment by a red and black star among horn-bearing and all aligned species, respectively.
(TIF)

Table S1 **Breed origin of case-control design, carriers and semi-random samples.** Breed names, abbreviations, geographic origin and numbers of genotyped samples are listed for each group within breed. The four groups are homozygous polled cases (PP), heterozygous polled carriers (Pp), horned controls (pp) and the semi-random sample (R) adjusted by the diversity panel.
(PDF)

Table S2 **Specification of the samples origin.** Name of the samples supplier, affiliation, sampled breed(s), sampled tissue and primary reason for tissue sampling. This table is part of Acknowledgments and Ethics statement.
(PDF)
