# Supplementary figures and images for: Bovine Polledness – An Autosomal Dominant Trait with Allelic Heterogeneity
Source: PLoS One. 2012 Jun 21;7(6):e39477. doi: 10.1371/journal.pone.0039477 (PMC3380827; doi:10.1371/journal.pone.0039477)

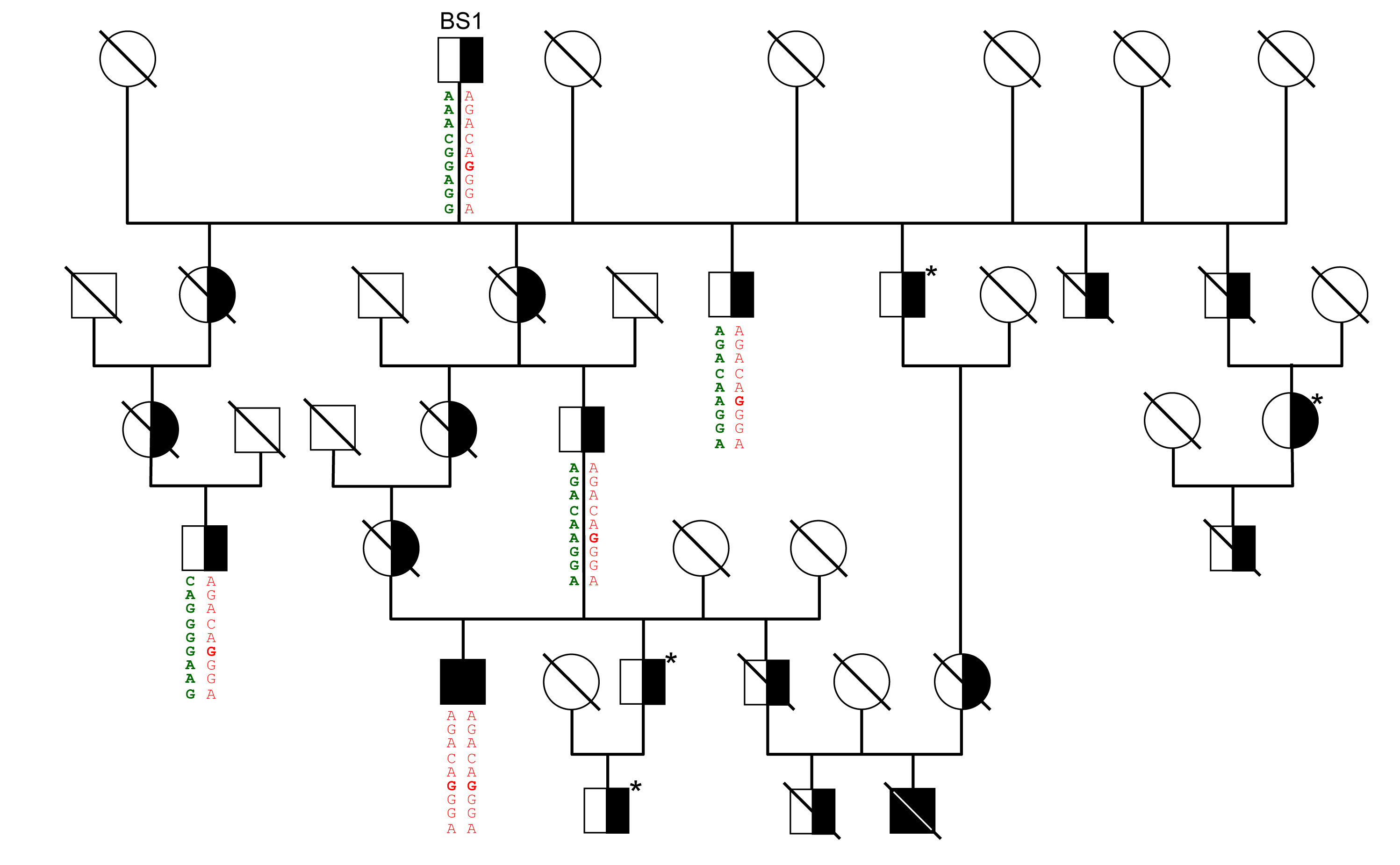

Supplement: Figure S1 — The pedigree chart of most polled Braunvieh. All declared polled Braunvieh bulls are descendents of the well-known American Brown-Swiss bull BS1 which is founder of polledness in Brown-Swiss/Braunvieh cattle population. The case individuals (PP) are represented by solid circles (females) and squares (males); declared carriers by half-filled symbols; not sampled individuals are marked with a diagonal line. The haplotype associated with polledness (red letters) of the four genome-wide genotyped bulls can be traced back to the same carrier bull BS1. This pedigree includes also four polled Braunvieh animals (*) genotyped only for candidate mutations. All sampled PP animals were genotyped as PC/PC and all Pp as PC/prs. (TIF) [file pone.0039477.s001.tif]

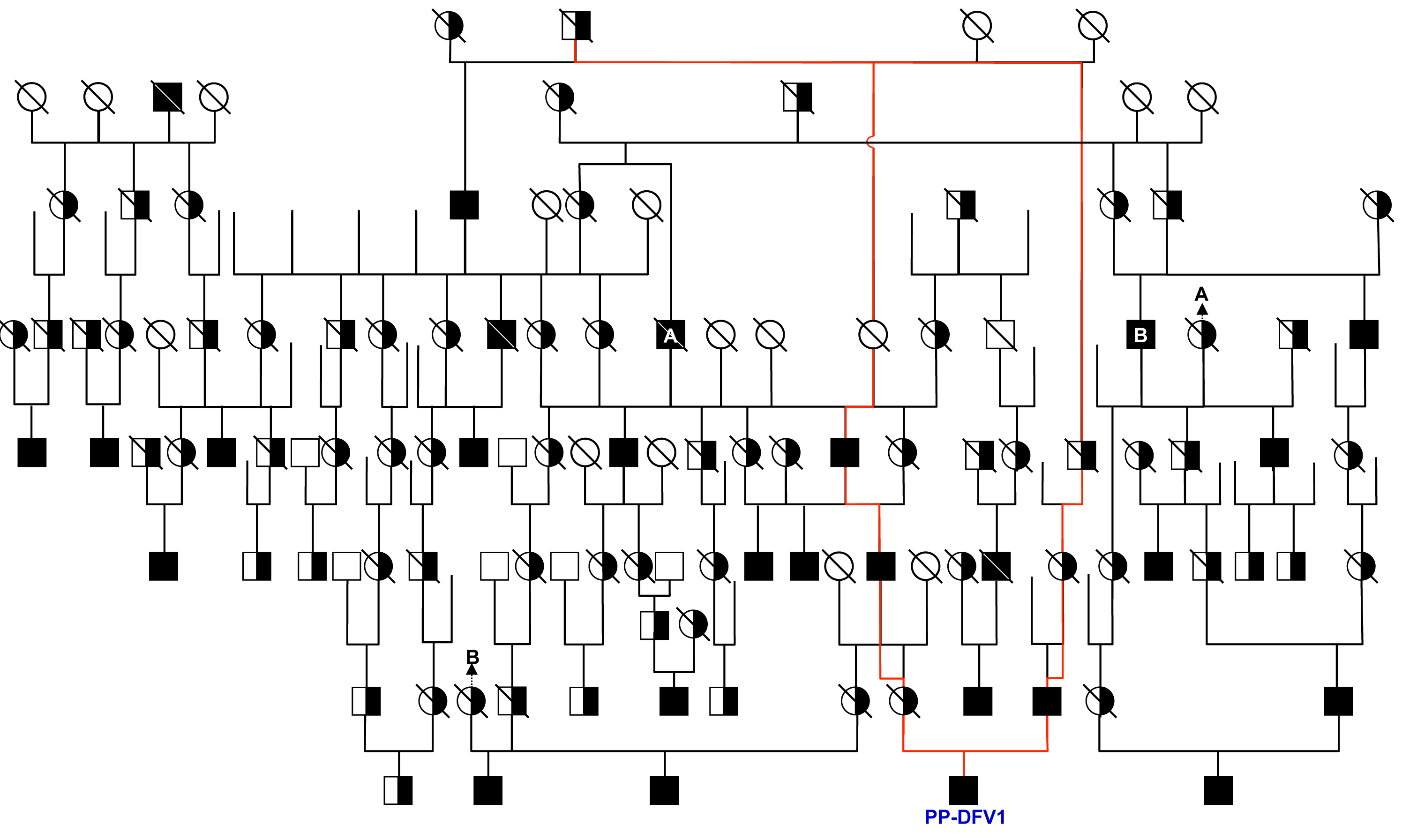

Supplement: Figure S2 — The pedigree chart of all sampled Fleckvieh bulls composing case and carrier group in Table S1. The case individuals (PP) are represented by solid circles (females) and squares (males); declared carriers by half-filled symbols; not sampled individuals are marked with a diagonal line. To reduce complexity of the pedigree not all relationships were presented. At two positions (A and B) there are relationships to important sires indicated. The inbreeding of the Fleckvieh bull (PP-DFV1) chosen for re-sequencing is superimposed by red lines. All sampled Fleckvieh PP animals bear two copies of the common haplotype (AGACAAGGA) and were genotyped as PC/PC. All Pp bear one copy of the common haplotype and were genotyped as PC/prs. No recombination was detected in the common haplotype. (TIF) [file pone.0039477.s002.tif]

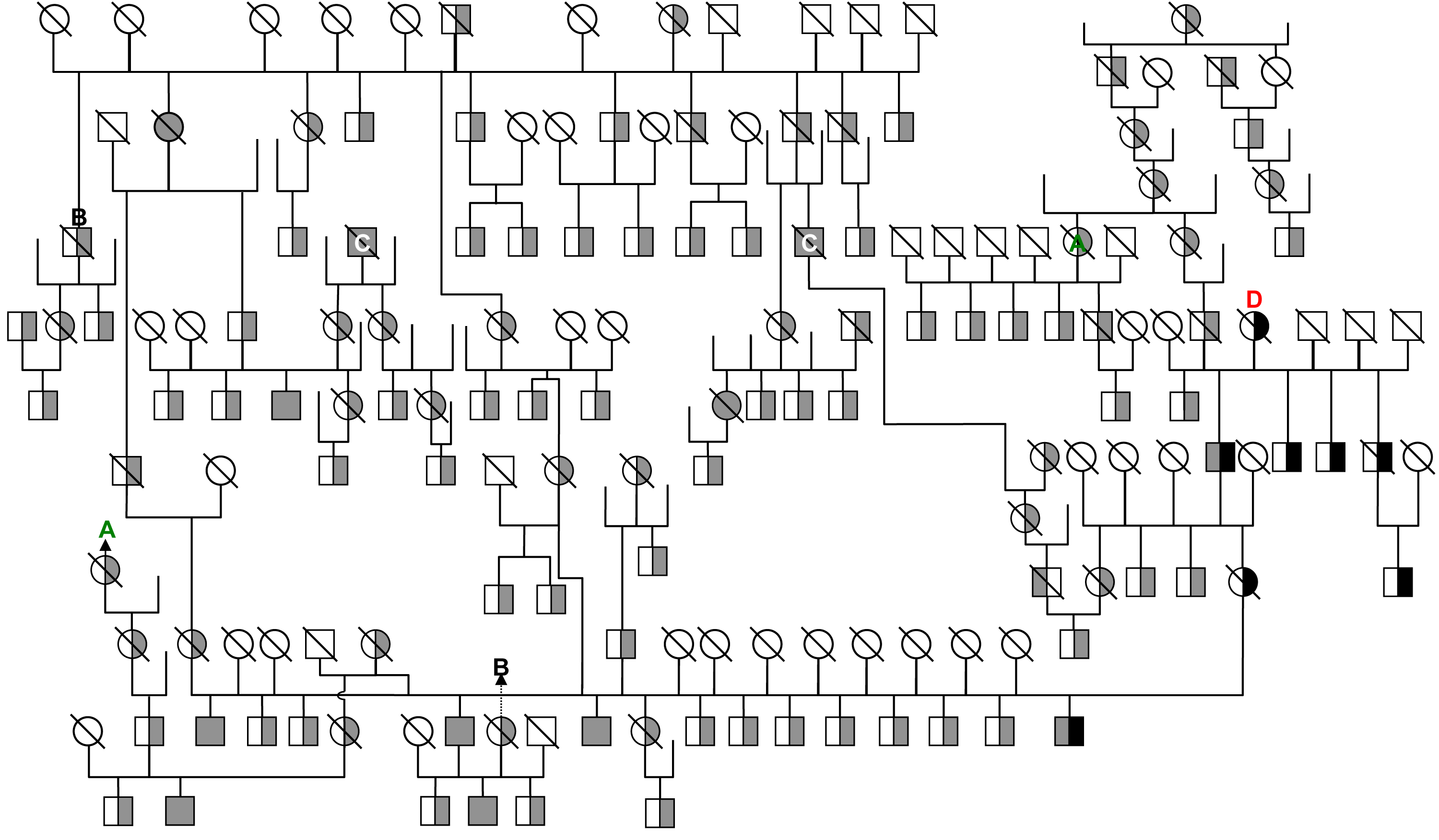

Supplement: Figure S3 — The pedigree chart of all sampled Holstein bulls composing case and carrier group in Table S1. The case individuals (PP) are represented by solid circles (females) and squares (males); declared carriers by half-filled symbols; not sampled individuals are marked with a diagonal line. To reduce complexity of the pedigree not all relationships were presented. At positions A, B and C there are relationships to important sires indicated. The founder of the Celtic polledness is indicated by D. The carriers and cases of Friesian and Celtic polledness are filled with gray (genotyped as PF/prs and PF/PF) and black color (genotyped as PC/prs and PC/PC), respectively. Two heterogeneous polled bulls were genotyped as PC/PF. At the SNP-Chip level all sampled Holstein PP and Pp animals bear two or one copy of the common haplotype (AGACAAGGA) and there was no recombination detected. (TIF) [file pone.0039477.s003.tif]

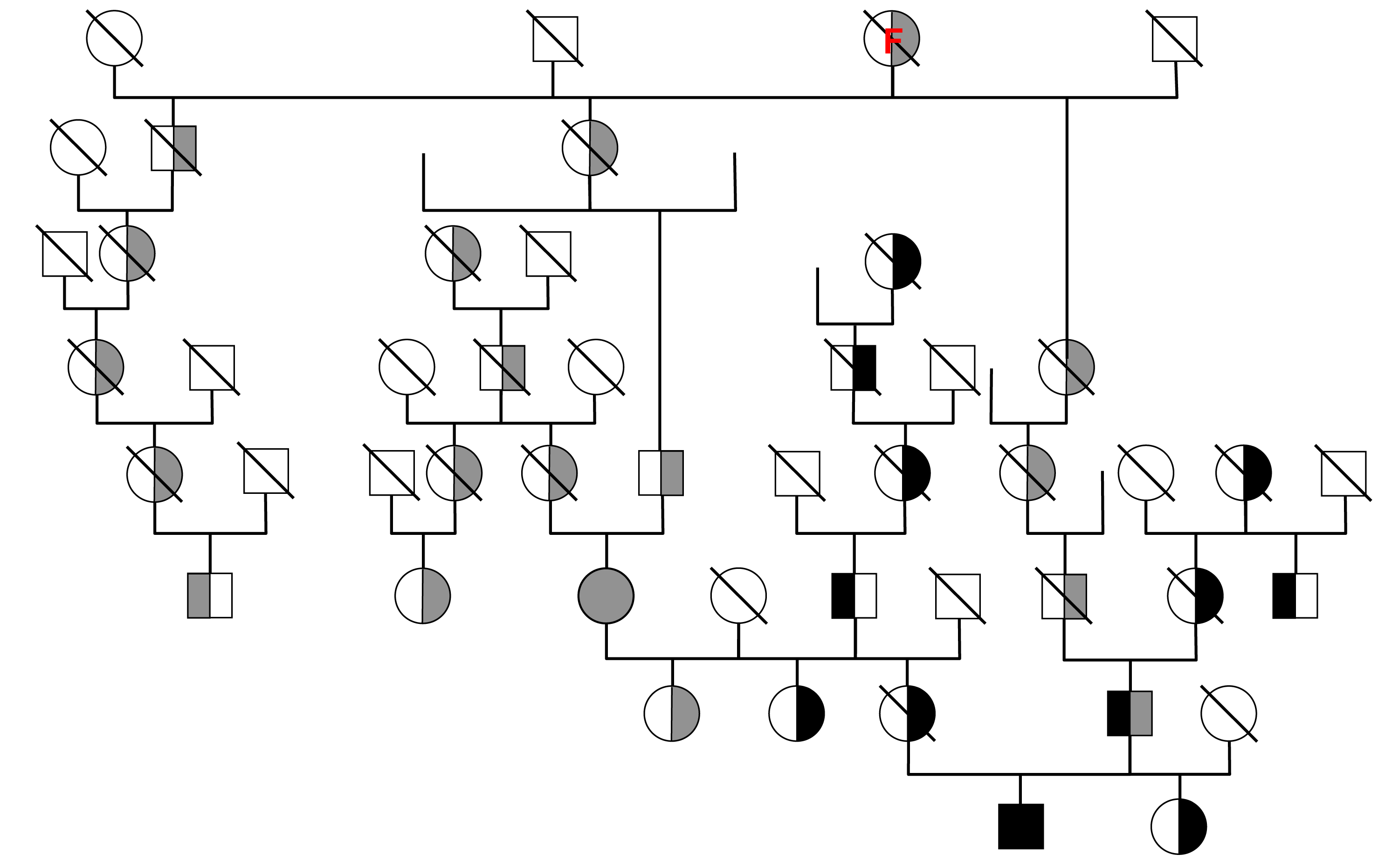

Supplement: Figure S4 — The pedigree chart of all sampled case and carrier Jersey bulls. The case individuals (PP) are represented by solid circles (females) and squares (males); declared carriers (Pp) by half-filled symbols; not sampled individuals are marked with a diagonal line. The founder of the Friesian polledness in Jersey breed is indicated by F. The carriers and cases of polledness with Friesian and Celtic origin are filled with gray (genotyped as PF/prs and PF/PF) and black color (genotyped as PC/prs and PC/PC), respectively. Two heterogeneous polled bulls were genotyped as PC/PF. At the SNP-Chip level all sampled Jersey PP and Pp animals bear two or one copy of the common haplotype (AGACAAGGA) and there was no recombination detected. (TIF) [file pone.0039477.s004.tif]

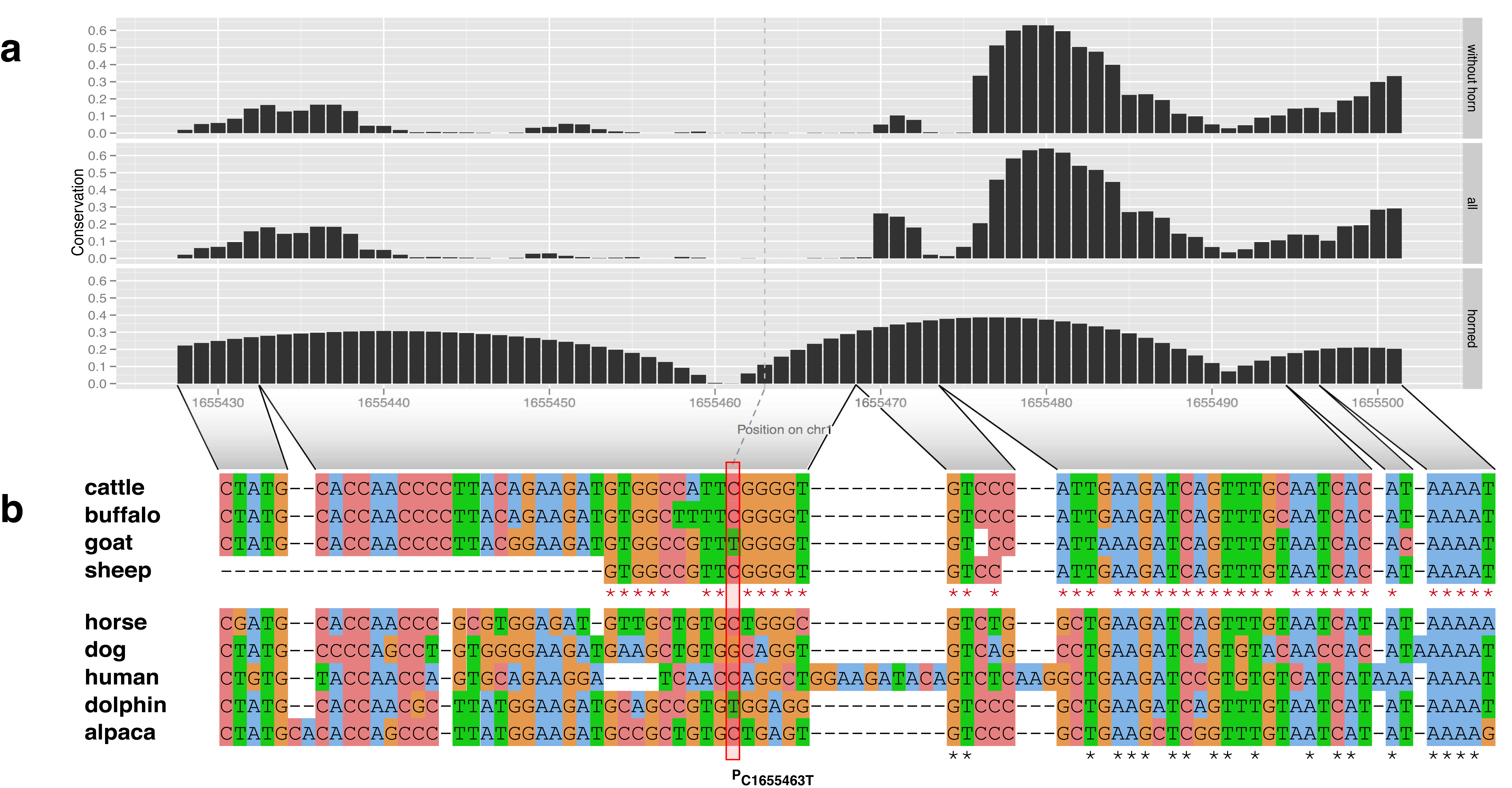

Supplement: Figure S5 — Across-species conservation for variant PC1655463T. (a) The sequence conservation around the position of the candidate mutation PC1655463T among animals without horn, horn-bearing and among all animals are represented as PhastCons scores, calculated from the underlying multi-species alignment in plot b. (b) Sequence identity among horn-bearing animals is denoted with red stars and black stars are used for identity in all aligned species. The position of the candidate DNA sequence variant is outlined by a dashed black line and is highlighted in the multi-species alignment by a red frame. (TIF) [file pone.0039477.s005.tif]

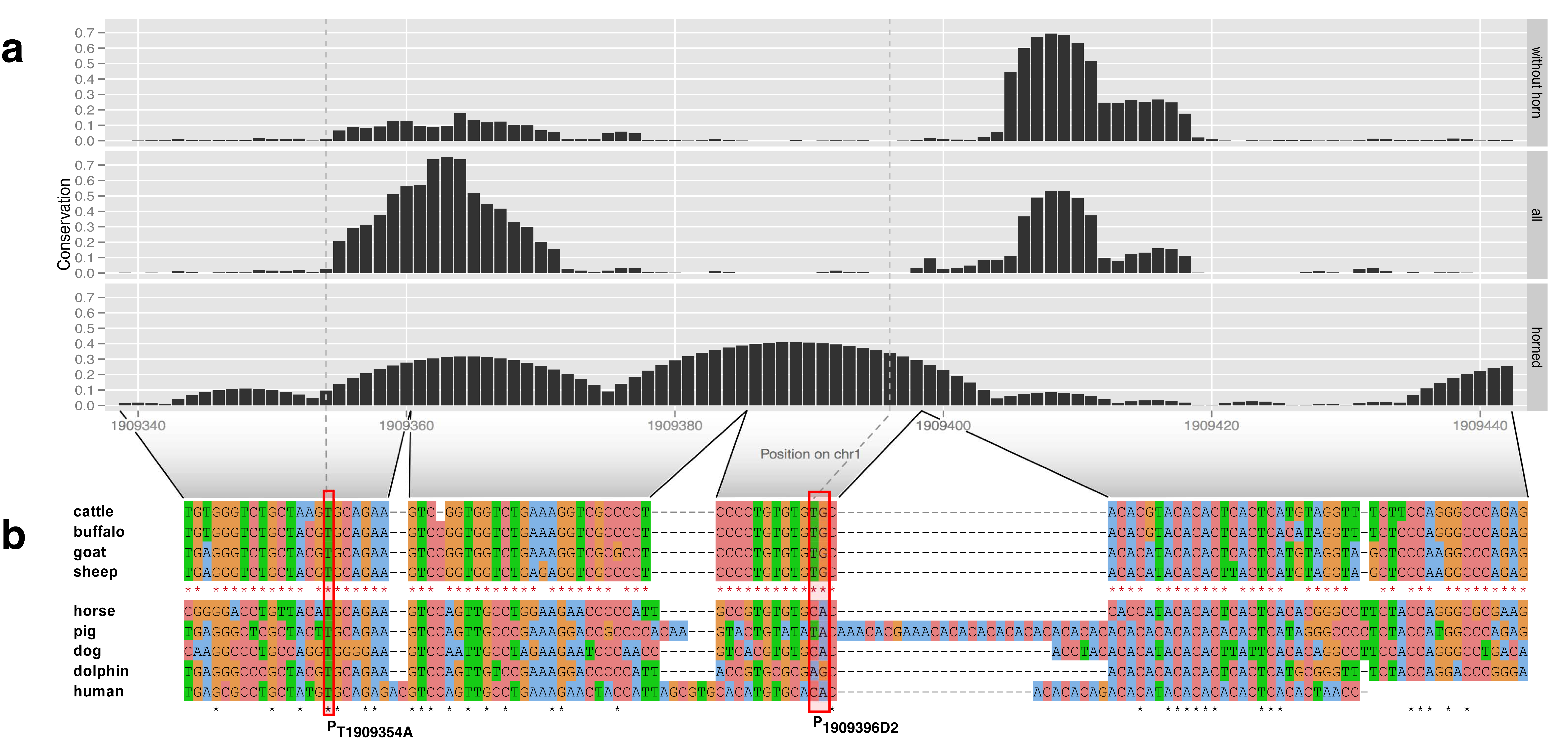

Supplement: Figure S6 — Across-species conservation for variants PT1909354A and P1909396D2. (a) The PhastCons conservation scores of the sequence around the two DNA sequence variants PT1909354A and P1909396D2 are shown. Both variants are outlined with black dotted lines. (b) Sequence identity is marked in the multi-species alignment by a red and black star among horn-bearing and all aligned species, respectively. (TIF) [file pone.0039477.s006.tif]
