# Supplementary material for: Bovine Polledness – An Autosomal Dominant Trait with Allelic Heterogeneity
Source: PLoS One. 2012 Jun 21;7(6):e39477. doi: 10.1371/journal.pone.0039477 (PMC3380827; doi:10.1371/journal.pone.0039477)
Supplement: Table S1 — Breed origin of case-control design, carriers and semi-random samples. Breed names, abbreviations, geographic origin and numbers of genotyped samples are listed for each group within breed. The four groups are homozygous polled cases (PP), heterozygous polled carriers (Pp), horned controls (pp) and the semi-random sample (R) adjusted by the diversity panel. (PDF) [file pone.0039477.s007.pdf]

No	Breed	Abr.	Origin	PP	Pp	pp	R
1	Anatolian black	ABB	Anatolia	-	-	10	36[*]
2	Barrosã	BAR	Portugal	-	-	10	14[*]
3	Blanc Bleu Belge	BBB	Belgium	-	-	20	46
4	Blonde d'Aquitaine	BAQ	France	1	1	1	5
5	Braunvieh	BBV	Germany	1	4	20	56
6	Charolais	CHA	France	1	3	1	10
7	Crossbred	HF-FV	Germany	-	-	-	293[#]
8	Fjall cattle	FJL	Sweden	1	-	-	-
9	Galloway	GLW	Scotland	45	-	-	13
10	Gelbvieh	FGV	Germany	-	-	-	2
11	German Angus	DAN	Scotland-Germany	76	3	-	18
12	German Fleckvieh	DFV	Germany	24	17	30	203
13	Glenvieh	GLV	Germany	-	-	-	1
14	Hereford	HER	England / Wales	1	-	-	-
15	Highland cattle	HGL	Scotland	-	-	-	1
16	Hinterwaelder	HWD	Germany	-	-	-	3
17	Holstein-Friesian	HF/RH	Germany	8	38	30	238[#]
18	Istrian cattle	HRI	Croatia	-	-	-	40[*]
19	Jersey	JY	Jersey	1	7	1	52[#]
20	Lekbibaj Cattle	LKB	Albania (north)	-	-	10	37[*]
21	Limousin	LIM	France	2	7	1	30
22	Maronesa	MAR	Portugal	-	-	10	14[*]
23	Murnau-Werdenfelser	MWF	Germany	-	-	-	3
24	Norwegian Red	NOR	Norway	1	6	8	5[#]
25	Pinzgauer cattle	PNZ	Austria	-	-	-	50[#]
26	Podolian cattle	HRP	Croatia	-	-	-	49[*]
27	Prespa Cattle	PRB	Albania (south)	-	-	10	19[*]
28	Sayaguesa cattle	SYG	Spain	-	-	-	2[*]
29	Vorderwaelder	VWD	Germany	-	-	-	20
30	Wagyu Cattle	WGY	Japan	-	-	-	2
31	Witrug	WTG	Netherlands	-	3	-	-
	ALL			162	89	162	1262

[#]Random animals sampled from target breeds.
[*] Random animals from cattle diversity panel.
