# Supplementary material for: Bovine Polledness – An Autosomal Dominant Trait with Allelic Heterogeneity
Source: PLoS One. 2012 Jun 21;7(6):e39477. doi: 10.1371/journal.pone.0039477 (PMC3380827; doi:10.1371/journal.pone.0039477)
Supplement: Table S2 — Specification of the samples origin. Name of the samples supplier, affiliation, sampled breed(s), sampled tissue and primary reason for tissue sampling. This table is part of Acknowledgments and Ethics statement. (PDF) [file pone.0039477.s008.pdf]

No	Name	Affiliations	Breeds	Samples	means of access	Primary reason for tissue sampling
1	K.H. Göpel	Göpel Genetik GmbH, Germany	NOR, FJL, HER, WTG, JY, GLV, RH/HF, LIM, CHA, BAQ, DFV, BBV, BBB	Semen	Donated 85% and purchase	Routine artificial insemination
2	Dr. A. Scholz	LVG, Oberschleißheim, Germany	HF-FV, MWF, FGV, BBV, HF/RH, FV, JY	Blood	Donated	Routine IBR diagnostics and ruminant metabolism control
3	M. Schricker	FVB, Ansbach, Germany	GLW	Blood, hair root	Donated	Routine paternity test
4	Dr. J. Eder	Hochschule Weihenstephan-Triesdorf, Germany	DAN	DNA	Donated	PhD study of Dr. J. Eder
5	Dr. A. Medugorac	TU-Munich, Germany	ABB, PNZ	DNA	Donated	PhD study of Dr. A. Medugorac
6	Dr. J. Ramljak<br>Dr. A. Ivankovic	University of Zagreb, Croatia	HRP, HRI	DNA	Donated	PhD study of Dr. J. Ramljak
7	Dr. K. Kume	NC of FAnGR, Tirana, Albania	PRB, LKB	DNA	Donated	Phylogenetic studies (ERFP Project).
8	Dr. H.P. Grünenfelder	SAVE, St. Gallen, Switzerland	PRB, LKB	DNA	Donated	Phylogenetic studies (ERFP Project).
9	Dr. V. Cadavez	CIMO, Braganca, Portugal	BAR, MAR	DNA	Donated	Master thesis advised by Dr.V. Cadavez
10	Dr. E.D. Hegemann	Hegemann, Soest, Germany	SYG	Blood	Donated	Routine IBR diagnostic.
11	H. Zuchtriegel	BSG, Greifenberg, Germany	FV, BBV, HF, CHA, LIM, BAQ and WGY	Semen	Donated	Routine artificial insemination
12	L. Eule	AgrarCenter Erzgebirge GmbH, Schwarzenberg, Germany	LIM	Semen	Donated	Routine artificial insemination
13	N. Hartmann	Klöck-Hartmann GbR, Bidingen, Germany	JY	Blood	Donated	Routine IBR diagnostic.
14	M. Wild	Family Wild, Prittriching, Germany	JY	Blood	Donated	Routine IBR diagnostic.
15	E. Rosenberg	LfL, Grub, Germany	BBV, FV	Semen	Donated	Routine artificial insemination
16	Dr. G. Röhrmoser	ASR, München, Germany	BBV, FV	Semen	Donated	Routine artificial insemination
17	S. Rist	AHG Kempten, Germany	BBV	Semen	Donated	Routine artificial insemination
18	M. Kelz	RBG Memmingen, Germany	BBV	Semen	Donated	Routine artificial insemination
19	Dr. J Potthast	RUW, Münster, Germany	RH	Semen	Donated	Routine artificial insemination
20	Dr. B. Weber	Masterrind GmbH, Verden, Germany	HF, JY	Semen	purchase	Routine artificial insemination
21	Mrs. B. Brentrup	WWS GmbH, Altenberge, Germany	HF, JY	Semen	purchase	Routine artificial insemination
22	Mr. P Lobet	BBG, Ciney, Belgium	BBB	Semen	purchase	Routine artificial insemination
